# 811. A prospective pilot multicenter study of *Aspergillus* colonization in the nasal cavity as a surrogate marker of invasive aspergillosis among acute myeloid leukemia patients and allogeneic stem cell transplant recipients

**DOI:** 10.1093/ofid/ofad500.856

**Published:** 2023-11-27

**Authors:** Natnai Charoonrochana, Natini Jinawath, Pitak Santanirand, Atisak Jiaranaikulwanich, Aruchalean Taweewongsounton, Chavachol Setthaudom, Tananun Tanpaibule, Dootsadeephorn Surin, Pansachee Damronglerd, Wasithep Limvorapitak, Sirapat Rungwittayatiwat, Subencha Pinsai, Pimjai Niparuck, Porpon Rotjanapan

**Affiliations:** Faculty of Medicine Ramathibodi Hospital, Mahidol University, Bangkok, Krung Thep, Thailand; Faculty of Medicine Ramathibodi Hospital, Mahidol University, Bangkok, Krung Thep, Thailand; Ramathibodi Hospital, Mahidol University, Bangkok, Krung Thep, Thailand; Faculty of Medicine Ramathibodi Hospital, Mahidol University, Bangkok, Krung Thep, Thailand; Faculty of Medicine Ramathibodi Hospital, Mahidol University, Bangkok, Krung Thep, Thailand; Faculty of Medicine Ramathibodi Hospital, Mahidol University, Bangkok, Krung Thep, Thailand; Faculty of Medicine Vajira Hospital, Bangkok, Krung Thep, Thailand; Faculty of Medicine Vajira Hospital, Bangkok, Krung Thep, Thailand; Faculty of Medicine Thammasat University, Rochester, Minnesota; Faculty of Medicine Thammasat University, Rochester, Minnesota; Chao Phraya Abhaibhubejhr Hospital, Chachoengsao, Chachoengsao, Thailand; MEDICAL EDUCATION CENTER OF CHAOPHYA ABHAIBHUBEJHR HOSPITAL, phayathai, Krung Thep, Thailand; Faculty of Medicine Ramathibodi Hospital, Mahidol University, Bangkok, Krung Thep, Thailand; Faculty of Medicine Ramathibodi Hospital, Mahidol University, Bangkok, Krung Thep, Thailand

## Abstract

**Background:**

Invasive aspergillosis (IA) is a common opportunistic infection in patients with acute myeloid leukemia (AML) and allogeneic stem cell transplant (SCT) recipients. Identifying at–risk patients may improve clinical outcomes when universal prophylactic therapy is not possible in a resource-limited setting. *Aspergillus spp.* colonization has been listed as a potential risk for infection development among patients with hematologic diseases, but this has not been explored in real-life clinical practice.

**Objectives:**

To evaluate the correlation of *Aspergillus* spp. colonization and invasive aspergillosis development and to assess nasal wash galactomannan as a potential predictor for IA among AML patients and allogeneic SCT recipients.

**Methods:**

A prospective pilot study at four tertiary care hospitals in Thailand was conducted from April 2021 to January 2023. All AML individuals requiring induction chemotherapy and patients admitted for a conditioning regimen for allogeneic SCT were enrolled. Nasal wash fluid was obtained for galactomannan and fungal culture to assess *Aspergillus* spp. colonization before chemotherapy. The patient’s demographic and relevant data were retrieved. All patients were monitored for IA development and the need for antifungal use for IA treatment.

**Results:**

25 AML patients and nine allogeneic SCT recipients were included. A nasal wash fungal culture positive for *Aspergillus* spp. was identified in 3/34 patients. Eighteen (52.9%) patients were diagnosed with invasive aspergillosis after a six-month follow-up, 15/25 among AML patients, and 3/9 among allogeneic SCT. 8 patients (23.5%) had nasal wash galactomannan >0.46, and 5/8 patients had IA. The cut-off value of nasal wash fluid galactomannan of 0.46 provided a sensitivity of 55.56% and specificity of 78.57% in diagnosing IA in AML patients.

**Conclusion:**

The incidence of *Aspergillus* spp. colonization by the traditional culture-based method was low at treatment initiation and did not suggest future IA development in this study. However, nasal wash galactomannan may help predict future IA development in AML patients, but due to low sensitivity may limit its role in actual clinical practice at a larger scale. Further studies are needed to verify the best strategy to predict the risk of IA in this population.

**Disclosures:**

**All Authors**: No reported disclosures

